# Contraceptive efficacy of sperm agglutinating factor from *Staphylococcus warneri,* isolated from the cervix of a woman with inexplicable infertility

**DOI:** 10.1186/s12958-019-0531-6

**Published:** 2019-10-27

**Authors:** Neeraj Chandra Pant, Ravinder Singh, Vijaya Gupta, Aditi Chauhan, Ravimohan Mavuduru, Vijay Prabha, Prince Sharma

**Affiliations:** 1 Department of Microbiology, South Campus, Basic Medical Science (Block I), Panjab University, Sector 25, Chandigarh, 160014 India; 2 Department of Urology, PGIMER, Chandigarh, India

**Keywords:** *Staphylococcus warneri*, Sperm agglutinating factor, FITC, Mg^2+^-ATPase, Acrosome reaction, Contraceptive efficacy, Safety study

## Abstract

**Background:**

Voluntary control of fertility is of paramount importance to the modern society. But since the contraceptive methods available for women have their limitations such as urinary tract infections, allergies, cervical erosion and discomfort, a desperate need exists to develop safe methods. Vaginal contraceptives may be the answer to this problem, as these are the oldest ways of fertility regulation, practiced over the centuries. With minimal systemic involvement, these are also the safest. Natural substances blocking or impairing the sperm motility offer as valuable non-cytotoxic vaginal contraceptives. Antimicrobial peptides (AMPs) isolated from plants, animals and microorganisms are known to possess sperm immobilizing and spermicidal properties. Following this, in the quest for alternative means, we have cloned, over expressed and purified the recombinant sperm agglutinating factor (SAF) from *Staphylococcus warneri,* isolated from the cervix of a woman with unexplained infertility.

**Methods:**

Genomic library of *Staphylococcus warneri* was generated in *Escherichia coli* using pSMART vector and screened for sperm agglutinating factor (SAF). The insert in sperm agglutinating transformant was sequenced and was found to express ribonucleotide-diphosphate reductase-α sub unit. The ORF was sub-cloned in pET28a vector, expressed and purified. The effect of rSAF on motility, viability, morphology, Mg^++^-dependent ATPase activity and acrosome status of human sperms was analyzed in vitro and contraceptive efficacy was evaluated in vivo in female BALB/c mice.

**Results:**

The 80 kDa rSAF showed complete sperm agglutination*,* inhibited its Mg^2+^-ATPase activity, caused premature sperm acrosomal loss in vitro and mimicked the pattern in vivo showing 100% contraception in BALB/c mice resulting in prevention of pregnancy. The FITC labeled SAF was found to bind the entire surface of spermatozoa. Vaginal application and oral administration of rSAF to mice for 14 successive days did not demonstrate any significant change in vaginal cell morphology, organ weight and tissue histology of reproductive and non-reproductive organs and had no negative impact in the dermal and penile irritation tests.

**Conclusion:**

The Sperm Agglutinating Factor from *Staphylococcus warneri,* natural microflora of human cervix, showed extensive potential to be employed as a safe vaginal contraceptive.

## Background

The burgeoning population is a serious concern and contraception is considered the most accepted way of controlling it [1]. Female contraceptive methods include intrauterine devices (IUDs), barriers, sterilization, hormone-based treatment (oral/injectable) and insert contraceptives. Out of these, the most well-known are hormone-based contraceptives. Despite the fact that these have great contraceptive efficacy and reversibility, they have many side effects viz. spotting and irregular bleeding, weight increase, queasiness, and mood alterations; are irritating and exceptionally troubling sometimes, which lead to their discontinuation [2]. To some degree, distinctive yet similar issues lead to considerably less use of IUDs as they may lead to infection, heavy menstrual bleeding, dysmenorrhea, and pain during insertion [3]. Likewise, barrier methods of contraception are though effective but also suffer from certain side effects such as difficulty in insertion, allergic reactions to latex or polyurethane, vaginal irritation, cervical erosion, urinary tract infections and rarely toxic shock syndrome, if the device is left set up for a really long time [4]. Sterilization is an excellent choice for women who truly want to terminate childbearing, but being an irreversible method, there is evidence of some regret, particularly among women sterilized at younger ages [5]. Every contraceptive method in use today has shortcomings, and collectively they leave major voids in the ability of people to control fertility safely, effectively, and in culturally acceptable ways throughout their reproductive life. Therefore, there is need to provide woman with safe and effective alternatives.

Commercially available vaginal contraceptive formulations contain non-ionic surfactants such as nonoxynol-9 (N-9), as an active ingredient that causes irreversible immobilization of human spermatozoa but affects vaginal microflora and the epithelial cells when used frequently [6]. Non-invasive contraceptive agents with spermicidal activity are acrylophenones, vanadocenes, gel microemulsions (GM-4, GM-44), sylidines, thymols, and isoxazoles/isoxazolines [7]. Amongst the natural products, saponins isolated from the fruit pericarp of *Sapindus mukorossi* [8]*,* magainin-A from the skin of the African clawed frog *Xenopus laevis* [9, 10] nisin- a bacteriocin produced by *Lactococcus lactis* [11–13] and subtilosin from *Bacillus subtilis* and *B. amyloliquefaciens* possess good spermicidal activity [14].

Recombinant proteins such as heat labile enterotoxin subunit B genetically linked with hCG-β chain [15], recombinant bonnet monkey zona pellucida (ZP1) conjμgated to diphtheria toxoid (used to immunize female baboons) [16] and sperm specific antigen, NZ1, have been reported to prevent pregnancy [17]. Also, various microorganisms reported to immobilize or agglutinate spermatozoa are *Escherichia coli* [18], *Chlamydia trachomatis* [19], *Mycoplasma genitalium* [20], *Ureaplasma urealyticum* [21], *Staphylococcus aureus* [22] and *Candida albicans* [23]. Hence, bacterial proteins can be explored and developed as contraceptive agents.

In this work, *Staphylococcus warneri* (*S. warneri*)*,* isolated previously in our laboratory from the cervix of a woman with inexplicable infertility, was found to agglutinate human and mouse spermatozoa in vitro*.* Further, sperm agglutinating factor (SAF) was isolated and purified and was able to show complete sperm agglutination in vitro. However, as the gene responsible for sperm agglutinating activity was unknown and the production of SAF from wild type bacteria was very low, the present study was designed to identify the SAF and enhance its production by heterologous over expression and to further evaluate the efficacy of recombinant SAF as a contraceptive agent in a female mouse model.

## Methods

### Bacterial strains and plasmid

*S. warneri* isolated from the cervix of a woman with inexplicable infertility, showed sperm agglutinating activity and was identified by Matrix-assisted laser desorption/ionization (MALDI) Microflex LT mass spectrometer [24]. It was maintained in Brain Heart Infusion broth. Plasmid pSMART, expression vector pET28a and *Escherichia coli* (*E. coli*) DH10β and BL21 (DE3) strains were used for the cloning and expression of recombinant protein.

### Sperm-bacteria interaction for sperm agglutinating activity

Sperm agglutinating activity was carried out as described earlier by Pant et al. [24]. Breifly, *S. warneri* was grown in Luria Broth (LB) at 37 °C/180 rpm for 72 h, following which it was centrifuged at 10,000 xg for 10 min at 4 °C. The supernatant was passed through a 0.22 μm Millipore filter to ensure that it was cell free. The bacterial cells so obtained were washed twice with sterile PBS. Equal volumes of semen sample (40 × 10^6^ spermatozoa ml^− 1^), whole cell culture or washed cells (10^7^ cells ml^− 1^) or cell free supernatant were mixed and incubated at 37 °C for 0, 15, 30, 60, 120 and 240 min and observed for agglutination at 400X magnification under light microscope. Sterile LB was used as control.

### Construction of genomic library

Chromosomal DNA was isolated and was partially restricted with HaeIII. The digest was run on a preparative gel and the agarose gel containing fragments (2–6 kb) was excised by sterile blade to extract DNA using the commercial QIAquick Gel Extraction kit (QIAGEN). Ligation was carried out with the linear pSMART vector (Lucigen) and transformed in electrocompetent *E. coli* DH10β cells (Lucigen) [24].

### Purification of recombinant sperm agglutinating factor (rSAF)

The recombinant cells were grown in LB-kanamycin for 72 h, washed twice with phosphate buffer saline (PBS) pH 7.4, sonicated and centrifuged at 10,000 xg for 20 min at 4 °C. Both the supernatant and pellet were checked for sperm agglutinating activity. Further, fractionation of supernatant was done with ammonium sulphate to get 20, 40, 60, 80 and 100% saturation and the precipitated and dialysed fractions were checked for activity. The bioactive fractions were further purified by Sephadex G-200 and checked for sperm agglutinating activity.

### Cloning, over-expression and purification of recombinant SAF

Chromosomal DNA of *S. warneri* was isolated [25] and used as template for PCR. Primers were designed by online tool ‘OligoEvaluator™’ having EcoRI and HindIII restriction sites in forward (5′-AAT**GAATTC**AATTGCACAACTTGTAC-3′) and reverse primer (5′-CGC**AAGCTT**ATGAAAACTATGGACGAG-3), respectively. PCR reaction was performed with initial denaturation at 94 °C for 3 min followed by 33 thermal cycles of denaturation at 95 °C for 1 min, annealing at 55 °C for 45 s, extension at 72 °C for 2 min and final extension at 72 °C for 10 min. The EcoRI and HindIII digested PCR product was ligated to similarly digested pET-28a and transformed into *E. coli* BL21 (DE3) by electroporation. Transformants were selected on LB-kanamycin agar plates and confirmed by polymerase chain reaction (PCR) [26]. For over-expression and purification, 500 ml of LB-kanamycin was inoculated with 2.5 ml of overnight grown inoculum of *E. coli* BL21 (DE3) containing pET-28a-SAF. When OD_600_ reached 0.8, Isopropyl β-D-1-thiogalactoside (IPTG) (0.5 mM) was added and incubated for 5 h at 37 °C/150 rpm. The cells were pelleted and suspended in 50 ml buffer (100 mM phosphate buffer, 300 mM NaCl, pH 8) containing 1 mg/ml lysozyme. The cell suspension was sonicated, centrifuged and loaded on Ni-NTA column. The column was washed with five column volumes of wash buffer (20 mM Tris–HCl, 500 mM NaCl, 20 mM imidazole, pH 8.0) to remove non-specific proteins. The bound SAF was eluted with buffer containing 20 mM Tris–HCl, 500 mM NaCl, 100 mM phosphate buffer, 250 mM imidazole, pH 8.0. Eluted fractions were collected and analyzed by 12% SDS PAGE [27]. Imidazole was removed by dialysis against PBS and protein concentration was estimated by Bradford kit and checked for sperm agglutinating activity.

### Binding of rSAF with spermatozoa

For this, 2 mg of purified protein was mixed with Fluorescence isothiocyanate (FITC), according to F/P ratio as per the instructions given in the kit (GeNei FITC Labelling Kit procured from Banglore Genei (India) Pvt. Ltd.). 100 μl of washed sperm suspension was incubated with 200 μl of FITC-rSAF at 37 °C for 1 h, post which 150 μl of 3% formaldehyde was added and again incubated at 37 °C for 1 h. After the completion of incubation period, the reaction mixture was washed thrice and suspended in 50 μl of PBS. A wet mount was prepared and observed under fluorescent microscope (1000X magnification).

### In vitro effect of rSAF on sperm morphology

Scanning electron microscopy (Joel Scanning Microscope, 6100, Jeol, Japan) was done to study the effect of rSAF on human spermatozoa morphology. Processing of samples was done according to the method described by [28].

### In vitro effect of rSAF on Mg^2+^-ATPase activity of spermatozoa

Mg^2 + −^ATPase activity of spermatozoa was estimated according to the protocol of [29] and [30]. Briefly, Tris-HCl (0.2 M, pH 7.6) washed spermatozoa (1 × 10^8^/mL) were sonicated at 50 Hz (10 cycles of 30 s with 1 min interval) at 4 °C. The reaction mixture for ATPase consisted of 200 μl each of Tris-HCl buffer (0.2 M, pH 7.6), Mg Cl_2_ (5 mM), ATP (6 mg mL-1), and sonicated sperm suspension. Different concentrations of rSAF (12.5, 25, 50 and 100 μg) were separately added and incubated at 37 °C for 1 h, post which the reaction was halted by adding 1 mL of cold 10% Tricholoroacetic acid (TCA) and then incubated at 4 °C overnight for protein precipitation. The control tubes contained all the components of the reaction mixture except that TCA was added in the beginning to halt the ATPase activity. Inorganic phosphorus (Pi) released was determined according to the method of [31]. One unit of ATPase was expressed as μmoles of the Pi released after 1 h of incubation.

### In vitro effect of rSAF on acrosome reaction of spermatozoa

The washed semen samples were resuspended in Ham’s F-10 medium (containing HEPES and 1% human serum albumin) were incubated for 3 h at 37 °C, post which spermatozoa were collected by centrifugation (500×g for 10 min) and their motility was assessed. The aliquots of motile spermatozoa (20× 10^6^ cells) incubated with either 0.1% Dimethyl sulfoxide (DMSO) (negative control) or 10 μM Calcium ionophore A23187 (positive control) or 25 μg of rSAF and incubated for 1 h at 37 °C. For the assessment of acrosomal status, a smear of sperm pellet was prepared on a glass slide, fixed with 95% ethanol for 30 min, air dried, washed with distilled water for 10 min and stained for 4 h with 25 mg/mL *Pisum sativum* agglutinin-Fluorescein isothiocyanate (PSA-FITC) in PBS (pH 7.4) at 4 °C. The slides were washed with distilled water, air dried and covered with 30 μL of anti-fading medium (50% v/v glycerol, 50% v/v distilled water, 25 mg/mL 1′,4-diazabicyclo [2] octane) and at least 100 spermatozoa were examined using a fluorescence microscope at 1000X. When more than half the head of a spermatozoon fluoresced brightly and uniformly, the acrosome was considered intact. Spermatozoa without fluorescence or with a fluorescing band limited to the equatorial segment were considered acrosome-reacted.

### In vivo contraceptive efficacy of rSAF

#### Animals

Sexually mature and randomly bred (5–6 week old male and 4–5 week old female) BALB/c mice were used. The animals were housed in polypropylene cages and were maintained under laboratory conditions (12:12, dark:light cycle) and fed with standard pellet diet and water ad libitum. All the animal procedures were designed for minimum pain and discomfort. The experimental protocols were approved by the Institutional Animal Ethics Committee of the Panjab University, Chandigarh, India vide letter no. PU/IAEC/S/15/72 and were performed in accordance with the guidelines of the Committee for the Purpose of Control and Supervision of Experiments on Animals (CPCSEA).

#### Fertility outcome

For the examination of contraceptive efficacy of rSAF, female BALB/c mice were used. In control group (*n* = 3), mice were administered with single intravaginal dose of 20 μl PBS. The test group was divided into 3 sub-groups with 5 mice in each sub-group receiving different concentrations of rSAF (2.5, 5 and 10 μg). Mice used for fertility studies were synchronised in their oestrous cycles by Whitten effect [32]. rSAF was deposited in the vagina while the mice were held in supine position for 1 min. All the animals were allowed to mate overnight with males of proven fertility (2:1). Next morning, the females were monitored for the presence of vaginal plug as confirmation of mating and the males were separated. The mice were examined for weight gain, abdominal distension and palpation of string of pearls and were kept under observation for entire gestation period.

#### Histological studies

From the above mentioned groups, one mouse each from control and test group were sacrificed on day 14 for histological evaluation. Reproductive organs were harvested, fixed in 10% formaldehyde for 24 h and then embedded in paraffin according to standard histological methods. Serial paraffin sections of 4 mm were stained with hematoxylin eosin and observed at 400 X magnification for any significant change in reproductive organs.

### Safety studies

#### Effect of r SAF on local toxicity

To appraise the local toxic effect of rSAF, six female BALB/c mice were divided into two groups viz. control and treated (3 animals each). rSAF at a dose of 5 μg/day/animal was administered intravaginally for 14 successive days. All animals were weighed on the first day of dosing and every seventh day thenceforth. The animals were checked at least twice daily for morbidity/mortality. Examination was also carried out for vaginal bleeding and discharges after 0.5 and 4 h after dosing. All the three animals of the control group received 0.1 ml of phosphate buffered saline (PBS). On day 15, animals from each group were sacrificed. The kidney, liver, spleen, ovary, uterus, and vagina were excised, fixed, and examined histologically [33].

#### Effect of rSAF on general health of mice

Female mice were divided into two groups (control and treated) of three animals each. rSAF was administered orally with the help of gavage once daily, at a dose level of 1 mg/kg body weight for 14 days to the treated group, at the same time the control group received PBS. On day 15, animals from each group were sacrificed. The non-reproductive (liver, kidney and spleen) and reproductive organs (ovaries and uterus) were removed and fixed in formalin. The sections were stained with hematoxylin and eosin, and evaluated for microscopic pathologic changes. Also, tissue somatic indices (TSI) (percent organ weight in relation to bodyweight) of reproductive and non-reproductive tissues of mice were recorded 24 h after the completion of 14 day oral administration of rSAF [12].

#### Primary dermal irritation test

Five micrograms and 25 μg of rSAF (test) or PBS (control) were applied topically once daily (50 μl/site/animal) for 5 consecutive days, to one non abraded and one abraded test site per mice. Each group consisted of 3 mice. The test site was throttled by covering each site with a gauze pad and over wrapping the site with plastic wrap. On day 6, dermal irritation was scored according to the Draize scoring system [34].

#### Penile mucosal irritation test

Five micrograms and 25 μg of rSAF or PBS (placebo) were applied directly (50 μl/site/animal) to the penis of three mature male BALB/c mice hourly for 4 h on 3 consecutive days. Each group consisted of three mice. All animals were observed for erythema and eschar formation prior to the application of the test material and at 1, 24, and 48 h after the last application [35]. The penises were dissected at the base of the pelvis (leaving the sheath intact), evaluated for gross pathology, and fixed in formalin. Sections from the fixed penises were blocked, sectioned, stained with hematoxylin and eosin, and evaluated for microscopic pathologic changes.

## Results

### *S. warneri* agglutinates human spermatozoa

**Fig. 1:**
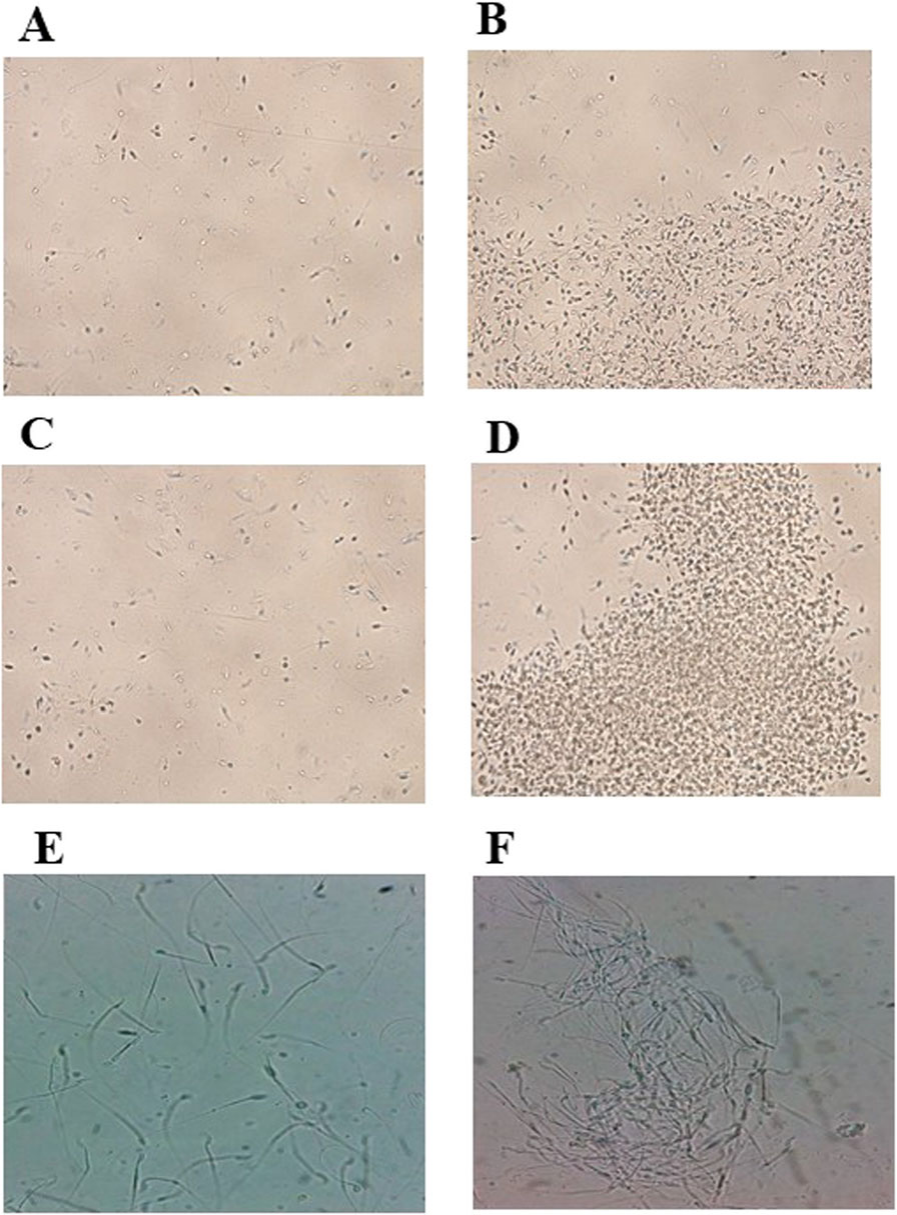
Representative photomicrograph (400X) showing sperm agglutination upon incubation with rSAF. Normal human (**a** and **c**) and mouse (**e**) spermatozoa incubated with PBS showed no agglutination; Human spermatozoa agglutinated with recombinant bacteria (**b**), human (**d**) and mouse (**f**) spermatozoa agglutinated with 50 μg of rSAF

A clinical isolate obtained from the *cervix* of a woman with inexplicable infertility agglutinated human spermatozoa in vitro (Fig. 1a, b). The isolate was identified as *S. warneri* by MALDI with log score 2.4. Whole culture and cells washed with saline agglutinated the spermatozoa whereas the culture supernatant did not. Sperm agglutination occurred in all possible orientation viz. in head-head, head-tail and tail-tail.

### Cloning of sperm agglutinating factor in *E. coli*

#### Construction of genomic library, screening of transformants and in silico analysis of SAF

In genomic library, a total of 3 × 10^5^ transformants were obtained and were screened for their sperm agglutinating activity (Additional file 1: Figure S1a, b). Recombinant plasmid from positive transformant was isolated and the insert was sequenced using vector primers SL1 and SR2 (forward and reverse) and a 2903 bp insert sequence was obtained (Additional file 1: Figure S2). ORF finder showed the presence of one ORF encoding ribonucleotide-diphosphate reductase-α sub unit. STRING analysis showed the proteins interacting with ribonucleotide-diphosphate reductase-α [24].

ProtParam online tool was used to predict physico-chemical parameters of ribonucleotide-diphosphate reductase-α (Additional file 1: Table S1). Secondary structure (Additional file 1: Figure S3) of ribonucleotide-diphosphate reductase-α is composed of alpha helices (291 amino acids), beta-sheets (106 amino acids) and random coils (304 amino acids). SWISS Model online tool was used to predict three dimensional structure of Ribonucleotide-diphosphate reductase-α sub unit using ribonucleotide reductase class 1b holocomplex R1E, R2F from *Salmonella typhimurium* (2bq1.1.B) with 42.39% identity as the closest template (Fig. 3a). Structure was validated by RAMPAGE and 631 (92.4%) residues were found in favourable, 35 (5.1%) in disallowed and 17 (2.5%) in outlier region (Additional file 1: Figure S4) showing its good quality stereochemical structure.

#### Expression and purification of rSAF under native conditions

**Fig. 2:**
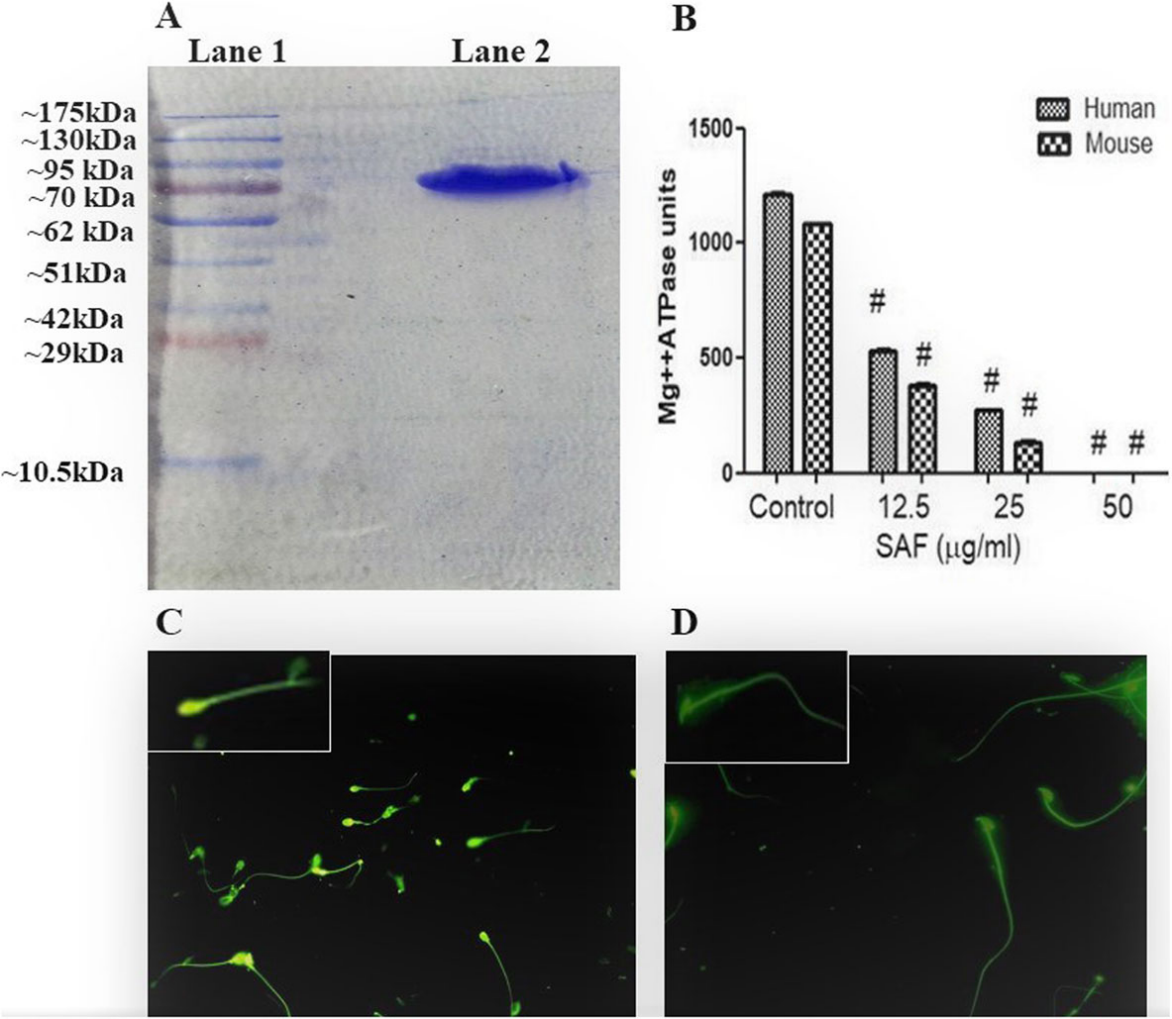
**a** Estimation of molecular weight of recombinant SAF. Lane 1: Pink plus Protein molecular weight marker; Lane 2: Purified recombinant SAF. **b** Inhibition of Mg^++^ dependent ATPase activity of human spermatozoa and mouse spermatozoa upon incubation with rSAF (0-50 μg). The results shown are mean ± S.D. of three observations, #*p* < 0.001. (H:human; M:mouse) (**c**) Visualization of FITC labeled recombinant rSAF incubated with human and **d** mouse spermatozoa by fluorescent microscopy (400X) showing rSAF binding to spermatozoa

**Fig. 3:**
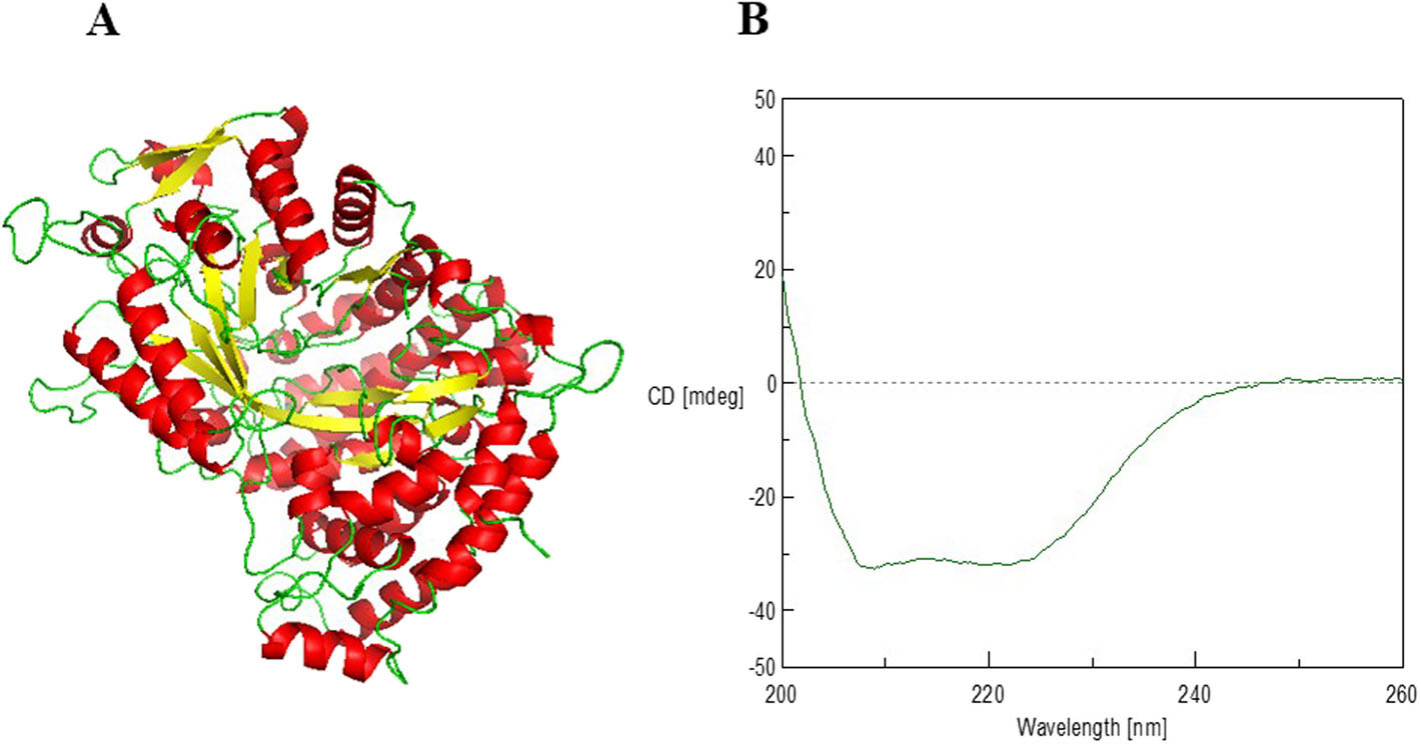
**a** 3-D structure of SAF modeled with SWISS-MODEL using homology modeling based on the template ribonucleotide reductase class 1b holocomplex R1E, R2F from *Salmonella typhimurium* (PDB ID: 2bq1.1.B) (**b**) CD spectra of recombinant SAF protein purified by Ni-NTA chromatography. The far UV CD spectrum of SAF protein analyzed by k2d2 software revealed that the secondary structure contained 49% α-helix, 17.8% β-strand and 33.2% random coil

Ribonucleotide-diphosphate reductase-α was sub cloned in pET-28a and expressed in *E. coli* BL21 (DE3) by induction with 1 mM IPTG at 37 °C for 5 h. Soluble protein was purified by Ni-NTA affinity chromatography under native conditions up to a concentration of 40 mg/L and resolved on SDS-PAGE (Fig. 2a). Quality of purified recombinant SAF was checked by Circular dichorism (Fig. 3b). Recombinant SAF (Ribonucleotide-diphosphate reductase-α sub unit) showed the sperm agglutinating activity when incubated with human and mouse sperms (Fig. 1c-f).

#### Binding of rSAF to spermatozoa

Fluorescence microscopy post treatment of sperm samples with FITC labelled rSAF revealed the presence of bright green fluorescence over the entire surface of spermatozoa depicting the binding of rSAF with spermatozoa and the fluorescence showed the presence of receptors on the entire surface of spermatozoa to which rSAF binds (Fig. 2c, d).

#### In vitro effect of rSAF on spermatozoa Mg^2+^-ATPase

In a dose-dependent manner, rSAF inhibited Mg^2+^-ATPase activity of human and mouse spermatozoa (Fig. 2b). At concentrations of 12.5 μg and 25 μg, Mg^2+^-ATPase activity declined from 1211.17 ± 9.3 units (control) to 532.93 ± 7.85 (44%) and 271.45 *±* 7.69 (22.4%) units, respectively. When rSAF was added at higher concentration i.e. 50 μg of SAF, no detectable Mg^2+^-ATPase activity could be observed. The effect of rSAF on the Mg^2+^-ATPase activity of mouse spermatozoa also displayed similar trend with rSAF at 50 μg showing maximum inhibition of Mg^2+^-ATPase activity. The units decreased from 1083.4 ± 9.07 (control) to 634.73 ± 4.79 (58.54%), 389.2 ± 3.16 (35.9%) and 144.51 ± 7.31 (13.33%) when incubated with rSAF at concentrations of 6.25, 12.5 and 25.0 μg, respectively.

#### In vitro effect of rSAF on human spermatozoan morphology and acrosome status

**Fig. 4:**
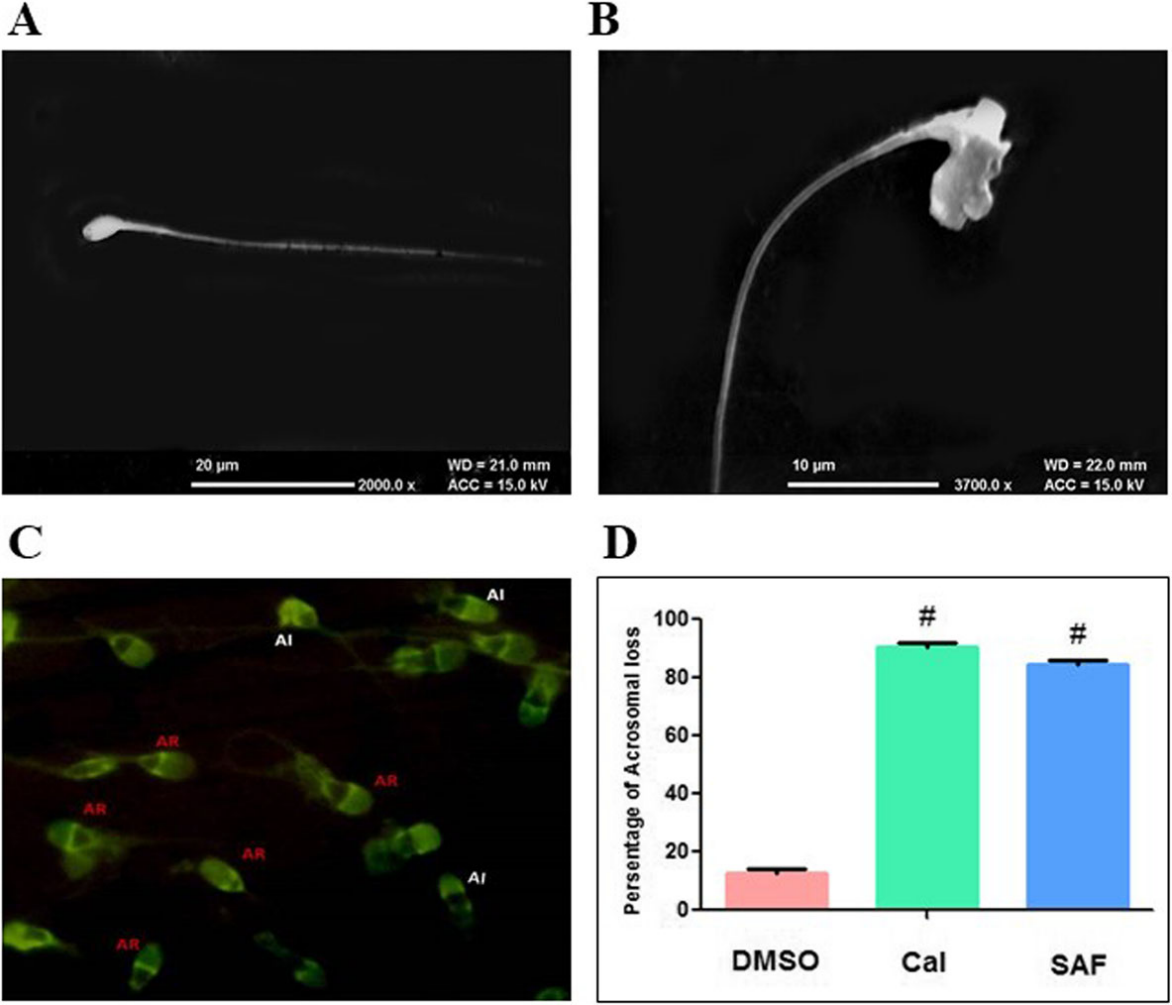
Scanning electron micrographs of human spermatozoan. **a** normal **b** treated with rSAF (100 μg,1 h) showing distortion of head. **c** Representative photomicrograph showing acrosome reacted (AR) and acrosome intact (AI) human spermatozoa as observed by fluorescence microscopy (400X) (**d**) Percentage of acrosome reacted spermatozoa after incubation with (0.1%) DMSO or (10 μg) Cal A23187 or SAF (25 μg). The values shown are mean ± SD of three observations, #*p* < 0.001

Scanning electron microscopy (SEM) showed that rSAF reacted with spermatozoa resulting in distortion of its head (Fig. 4a, b). Further, effect of rSAF (25 μg) on human sperm acrosome status was analysed and two patterns of fluorescence were observed. The spermatozoa with bright green fluorescence on more than half of the head indicated intact acrosomes (AI), whereas spermatozoa with only a fluorescing band at the equatorial segment were interpreted as acrosome-reacted (AR) (Fig. 4c). Upon incubation of spermatozoa with rSAF, it was observed that rSAF could lead to premature acrosome reaction (Ar) to the extent of 84.5 ± 1.8%. However, in case of negative control (DMSO) percentage of acrosome reacted spermatozoa was 12.66 ± 1.5%. The results obtained in case of rSAF were comparable to that induced by calcium ionophore (90.66 ± 1.60%), (positive control) (Fig. 4d).

#### Contraceptive efficacy of rSAF

**Fig. 5:**
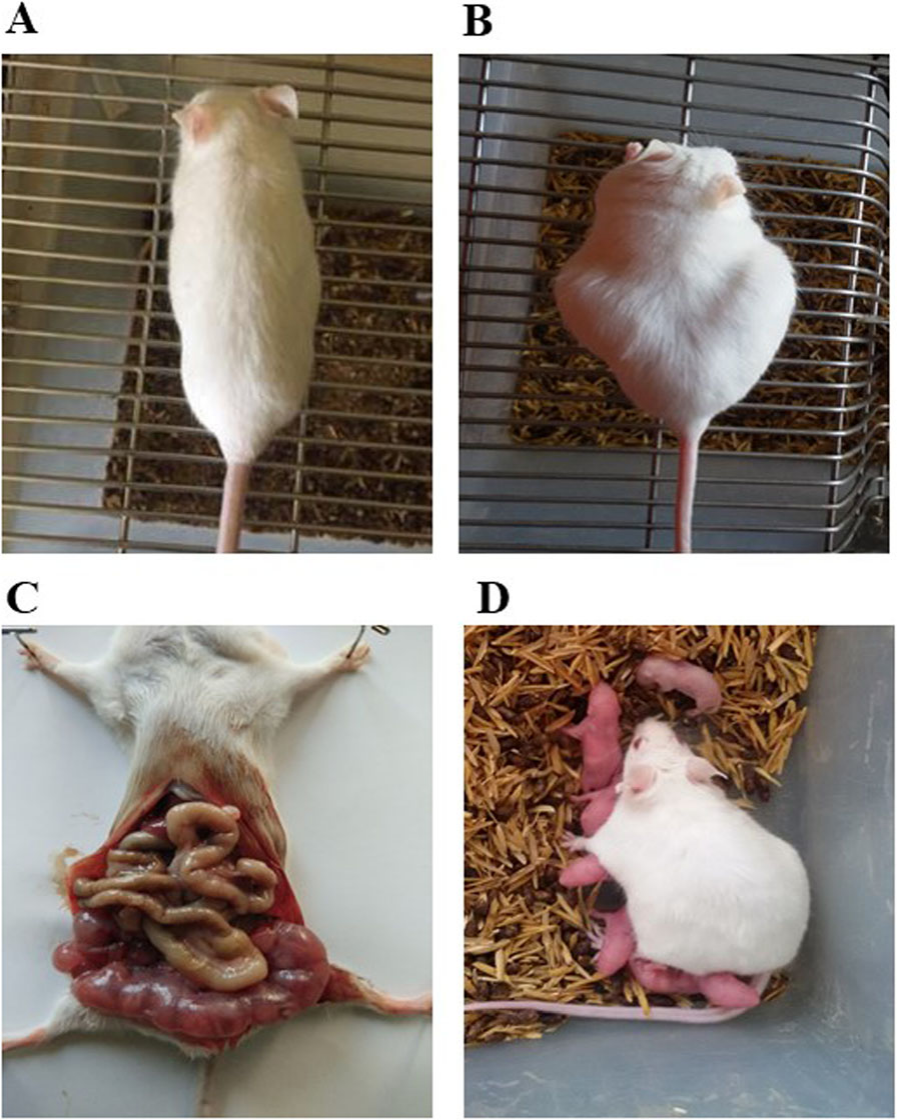
Representative photographs of pregnancy-related changes in female BALB/c mice instilled with PBS/2.5 μg rSAF (**a**) Day 0 of gestation (**b**) Day 22 showing abdominal distension, (**c**) with string of pearls on day 14 of gestation (**d**) delivery of pups at the end of gestation period

The control group mice (administered with PBS) displayed consistent weight gain, abdominal distension along with palpation of string of pearls and at the end of gestation period, delivered pups. rSAF when administered at lower concentration (2.5 μg) showed results comparable to control group (Fig. 5). However, all these changes were absent in group of mice receiving rSAF at concentrations ≥5 μg, indicating its excellent in vivo contraceptive efficacy (Table 1).

**Table 1:** Effect of rSAF on fertility outcome in female mice

rSAF dose instilled (μg/20 μl)	Number of female mice per group	Fertility outcome
PBS	3	3/3 (100%)
2.5 μg of rSAF	5	5/5 (100%)
5 μg of rSAF	5	0/5 (0%)
10 μg of rSAF	5	0/5 (0%)

#### Histology

**Fig. 6:**
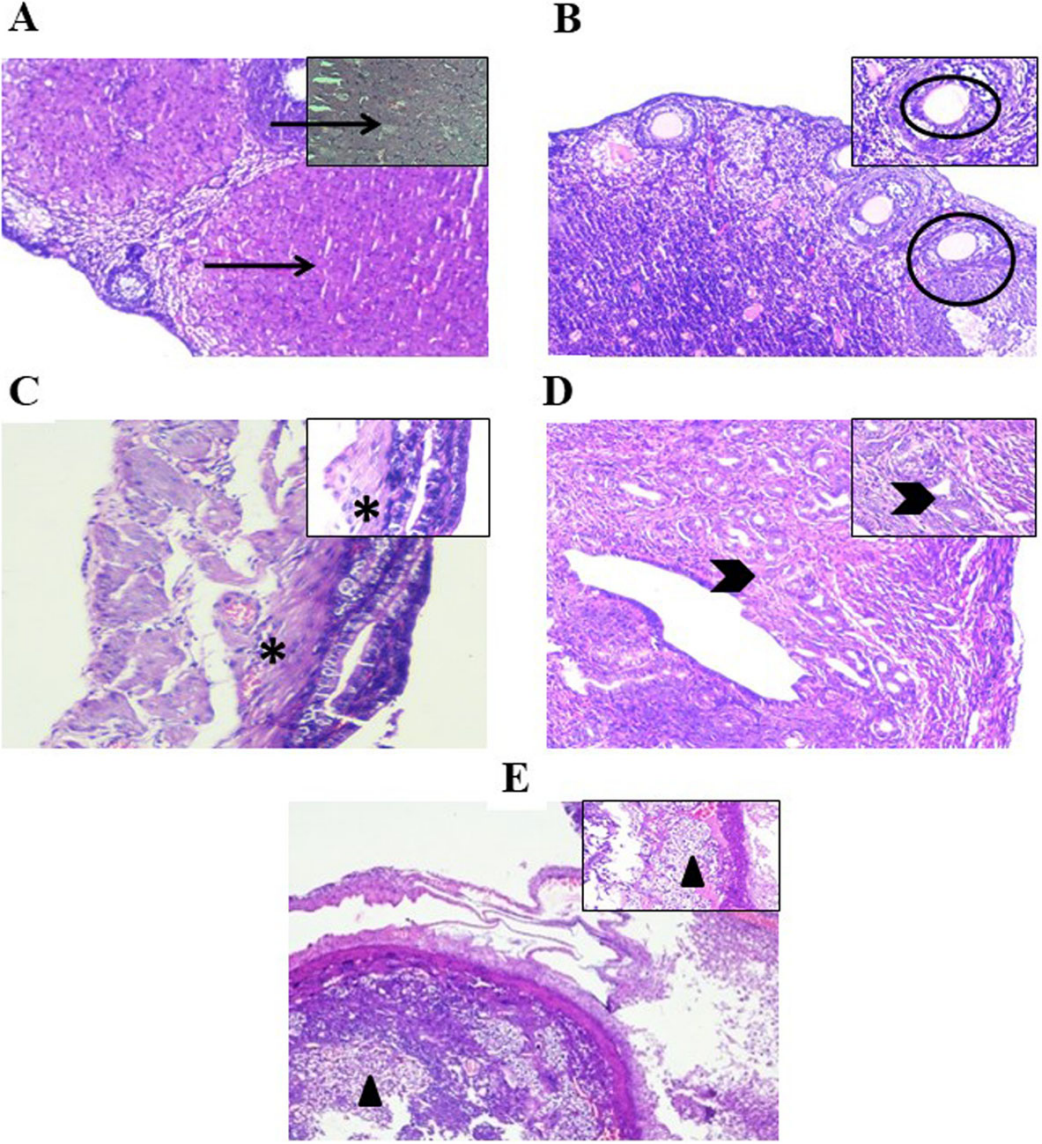
Histology of female reproductive organs on gestation day 14 in mice treated with PBS (controls: **a**, **c**, **e**) or 5 μg of recombinant SAF (tests: **b**, **d**). **a** Control ovary (arrow is showing the presence of corpus luteum); **c** control uterus (asterisk is showing the presence of deciduas E), **e**) placenta (triangle is showing the trophoblastic cells). rSAF treated (**b**) ovary (circle is showing graafian follicles), **d** uterus (arrow head is showing the normal muscles without any deciduas, as no pregnancy related changes were observed. Original magnification 100X, the predominant aspect is shown in the inset (400X)

Further, the histological evaluation of reproductive organs of mice was carried out to observe the pregnancy associated changes on days 0 and 14. In case of both control and rSAF treated groups, ovary and uterus showed normal tissue histology on day 0 before mating. However, on day 14 post mating, development of corpus luteum in ovaries was observed that indicated luteal phase were observed only in case of control mice and the group receiving lower concentration of rSAF i.e. 2.5 μg. In uterus, stromal decidualization and thickening of endometrium followed by proliferation and differentiation of uterine endometrium showed all the pregnancy related changes. All these pregnancy related alterations were absent in groups of mice instilled with higher concentrations rSAF viz. 5 μg and 10 μg (Fig. 6).

### Safety studies

#### Effect of rSAF on local toxicity in mice

Vaginal irritation studies performed in mice revealed no significant changes in mean body weight among the treatment groups (5 μg rSAF) or untreated groups (PBS). Also, no differences were observed among the treatment and control groups in terms of gross observations of the organs at necropsy and organ weights. Further, no change in the histology of reproductive organs and vital non-reproductive organs of mice was observed with respect to control mice (Additional file 1: Figures S5, S6). The mouse vagina was also studied for histopathological changes and no histological alterations could be attributed to rSAF. There was no edematous thickening of the submucosal layer or infiltration of polymorphonuclear leucocytes in to the mucosa. Thus, rSAF did not produce local toxicity in mice following vaginal application for a 14-day period.

#### Effect of rSAF on general health of mice and on tissue somatic indices

During the 14 day oral treatment, no behavioural changes (such as lethargy, aggression etc.) were observed in the treated mice. After the completion of 14 day oral treatment with rSAF, no change in the tissue histology of reproductive and non-reproductive organs of mice was observed in comparison to control mice. Further, the % TSI also showed no significant change in the weight of the organs of treated group in comparison to control group (Additional file 1: Table S1).

#### Dermal irritation test

No treatment related changes (edema, erytherma or eschar formation) were observed in any of the mice at 24 h, 48 h and 72 h examination points after application of rSAF for 5 consecutive days (Additional file 1: Figure S7). Therefore, rSAF was considered as non irritating to the skin.

#### Penile mucosal irritation test

Penile irritation studies in mice revealed no gross morphological or histopathological changes attributed to rSAF at 5 μg or 25 μg concentration (Additional file 1: Figure S8) in comparison to placebo.

## Discussion

According to the United Nations, the world population will reach 11.2 billion by the year 2100 [36] and contraception is the key solution to thwart this problem. Contraceptive methods to control the population are aplenty and many more are in pipeline. Condoms, intrauterine devices and oral contraceptives are successful tools available since long but they are associated with number of limitations, hence, there is an urgent need to develop safe, inexpensive and highly effective contraceptive methods. In this regard, sperm impairing agents or spermicides have come into light. Any sperm impairing agent, which immediately and irreversibly agglutinates or immobilizes the spermatozoa, not affecting developing foetus, non-irritating to penile and vaginal mucosa, non-toxic and not absorbed systemically could be a boon to field of contraception.

Various pathogens have been isolated from the semen of infertile and fertile patients interacting with the spermatozoa in terms of their agglutination and morphologic alterations [18–23]. These pathogens are causative agents of genitourinary infections and affect the functioning of spermatozoa by reducing their motility and damaging their structure [37]. Moretti et al. (2009) reported that *E. coli* elicits detrimental effect on spermatozoa such as swelling of mid-piece and tail invagination [38]. Also, they suggested the binding of *E. coli* on spermatozoa and its subsequent destruction in a two-step process [39]. Paulson and Polakoski, (1977) proposed a mechanism of sperm immobilization by *E. coli* and a factor excreted by *E. coli* that immobilized spermatozoa without agglutinating it [40]. Similarly, Diemer et al., (1996) reported that binding of *E. coli* results in inhibition of sperm motility via sperm agglutination [37]. Mannose has been found to interfere with binding of *E. coli* to spermatozoa but exact mechanisms of bacterial and sperm interactions have not been identified [41]. *S. aureus* has also been found as predominant flora in semen of infertile men and causes infertility by reducing sperm motility [42]. Emokpae et al. (2009) found *S. aureus* as major contributor in seminal infections [43]. Ohri and Prabha (2005) reported *S. aureus* to cause sperm agglutination and an unknown protein as sperm agglutinating factor from this bacterium has been proposed responsible for this phenomenon [44].

Role of microorganisms in impairing sperm motility is well known; hence microorganisms from the cervix of a woman with unexplained infertility were obtained and screened for sperm agglutinating activity in vitro. Clinical isolates showing positive sperm agglutinating activity were identified by MALDI. *S. warneri* was the organism impairing sperm function and is a close homologue of *S. aureus,* reported as sperm agglutinating agent [45]. *S. warneri* was evaluated for sperm agglutinating activity and it was found that whole culture and bacterial cells washed with saline were able to agglutinate the sperm but culture supernatant could not. Sperm agglutination occurred in head-head, head-tail and tail-tail orientations that shows the presence of receptors on whole sperm. Ribonucleotide-diphosphate reductase-α sub unit gene was identified by creating shotgun genomic library that was further over expressed and purified.

Motility, acrosome status and morphology are the main parameters to determine fertilization potential of spermatozoa. Any agent interferes with any of these parameters could be exploited as contraceptive. Therefore, all these three parameters were studied by incubating sperm with rSAF and it agglutinated sperm resulting in immobilization in vitro*.* Complete arrest of sperm motility has been reported within 20s using 100 μg SAF as compared to 400 μg nisin [12] and 1 mg magainin-A indicating higher efficiency of SAF [46]. Interestingly, Kaur and Prabha reported the irreversible effect of SAF on sperm motility as spermatozoa incubated with SAF remained immotile even after removing SAF from the reaction [47]. This showed the irreversibility of binding and possible cytotoxicity exerted by SAF. Moreover, incubation with SAF led to the complete loss of sperm viability at high concentrations within 20s showing the efficacy and rapidity of SAF mediated sperm damage. Interestingly, effects of SAF can be neutralized by anti-SAF antiserum to inverse the infertility. Kaur et al. (2013) raised the anti-SAF antibodies and found that in the presence of antibodies, sperm agglutination was blocked by inhibiting the binding of SAF to spermatozoa thus leading to conception in mouse model [48].

Motility is the most important character of spermatozoa required for the fertilization as immotile spermatozoa fail to meet oocyte and fertilization is inhibited. ATP provided by mitochondrian is required for sperm motility to power spermatozoa to the site of fertilization [49]. Cation dependent ATPases are responsible for the flagellar contractile processes and active transport [50]. Sperm moves due to ATP hydrolysis catalyzed by dyenin ATPase that is an Mg^2+^dependent enzyme located on axoneme [51]. There is a direct correlation between the sliding velocity and the quantity of dyenin arm present on the axoneme [52]. Therefore, inhibition of Mg^2+^dependent ATPase is an important parameter while studying sperm function as it is the primary sperm motility regulatory step. Hence, effect of rSAF on Mg^2+^dependent ATPase activity was analyzed and the results showed that rSAF acted as potent inhibitor of enzyme and decreased the activity in a concentration dependent manner. This inhibition of sperm Mg^2+^ dependent ATPase could be implicated as one of the mechanisms of impairing sperm motility by rSAF.

Premature acrosome reaction and acrosome reaction failure are crucial aspects of sperm function and are considered as important causes of infertility. The acrosome reaction is a receptor mediated exocytic process that involves outer acrosomal membrane and the sperm plasma membrane resulting in release of acrosomal enzymes required for fertilization [53]. The sperm with intact acrosome reaching the egg is required for fertilization that undergoes induced acrosome reaction on the surface of the zona pellucida [54]. Spermatozoa bind to zona pellucida and oocyte along with its surrounding cells release progesterone resulting in induction of acrosome reaction. Spermatozoa in cervix/vagina losing their acrosome prematurely lose their fertilization ability resulting in infertility [55]. Kaur et al. reported the inducing effect of SAF on premature AR and apoptosis in spermatozoa [56]. Therefore, acrosome status was analysed by incubating spermatozoa and rSAF that resulted in significantly higher rate of acrosome reaction and was comparable to CaI (positive control) when observed by fluorescent microscopy, proving its involvement in sperm damage. rSAF was found to induce premature AR in spermatozoa thus, decreasing its fertilization potential.

To understand the interaction between rSAF and spermatozoa, binding studies were carried out. When FITC labelled rSAF was incubated with human and mouse spermatozoa, fluorescence was observed over the entire spermatozoa indicating that receptors for rSAF are present on whole body i.e. sperm head, neck and tail. The uniform distribution of receptor on head, tail and body of spermatozoa resulted in mixed type of agglutination (head-tail, tail-tail and head-head). Further, intravaginal inoculation of rSAF in mice resulted in blockage of fertility was evident by absence of pregnancy related changes when observed apparently and histologically. Subsequent to assessing the contraceptive efficacy of the rSAF its toxicological impacts were likewise considered and rSAF was found to cause no toxicological effects at the contraceptive dose.

## Conclusion

The present study suggests that rSAF possessed admirable spermicidal activity in vitro and excellent contraceptive efficacy in vivo. Further, its high safety profile makes it a potential candidate to be developed as an effective vaginal contraceptive in future.

## Supplementary information

**Additional file 1.** Supporting data.

## Data Availability

All data generated or analysed during this study are included in this article.

## Abbreviations

AI: Intact acrosomes
AMPs: Antimicrobial peptides
AR: Acrosome-reacted
Ar: Premature acrosome reaction
DMSO: Dimethyl sulfoxide
IPTG: Isopropyl β-D-1-thiogalactoside
LB: Luria Broth
MALDI: Matrix-assisted laser desorption/ionization
PBS: Phosphate buffer saline
PCR: Polymerase chain reaction
Pi: Inorganic phosphorus
PSA-FITC: *Pisum sativum* agglutinin-Fluorescein isothiocyanate
rSAF: Recombinant sperm agglutinating factor
SAF: Sperm agglutinating factor
TCA: Trichloroacetic acid
