# Supplementary material for: Contraceptive efficacy of sperm agglutinating factor from Staphylococcus warneri, isolated from the cervix of a woman with inexplicable infertility
Source: Reprod Biol Endocrinol. 2019 Oct 27;17:85. doi: 10.1186/s12958-019-0531-6 (PMC6815424; doi:10.1186/s12958-019-0531-6)
Supplement: Supplementary file 1 — Additional file 1. Supporting data. [file 12958_2019_531_MOESM1_ESM.docx]

**Additional file 1**

**Contraceptive efficacy of sperm agglutinating factor from *Staphylococcus warneri,* isolated from the cervix of a woman with inexplicable infertility**

Neeraj Chandra Pant^1^, Ravinder Singh^1^, Vijaya Gupta^1^,Ravimohan Mavuduru^2^, Vijay Prabha^1^and Prince Sharma^1*^

^1^Department of Microbiology, Panjab University, Chandigarh, India

^2^Department of Urology, PGIMER, Chandigarh, India

*** Correspondence:** Prof. Prince Sharma, Department of Microbiology, South Campus, Basic Medical Science (Block I), Panjab University, Sector 25, Chandigarh, 160014, India

Telephone: +919815957903

e-mail: princess@pu.ac.in

Fax: 91-172-2541770


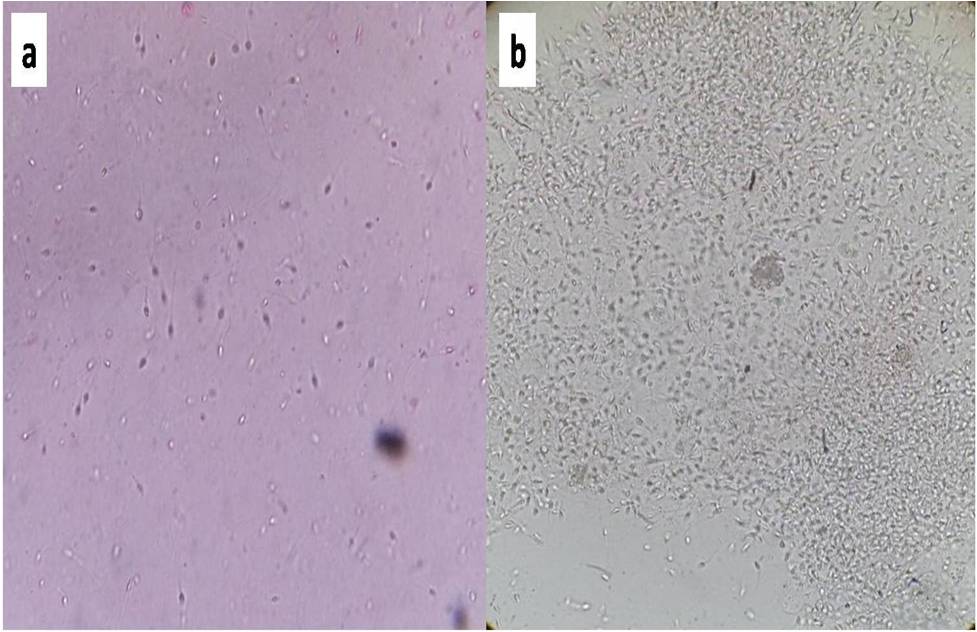


**Figure S1:**a) Normal human spermatozoa b) Agglutination of human spermatozoa (40×10^6^) upon incubation with 72 h old culture of *S.warneri* (1×10^8^cfu) for 30 min at 37°C(400X).

**
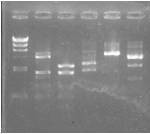
**

**Figure S2:** Plasmid isolated from positive transformant: M: λ/Hind III marker; Lane 1:recombinant pSMART plasmid


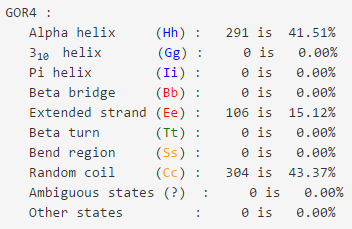


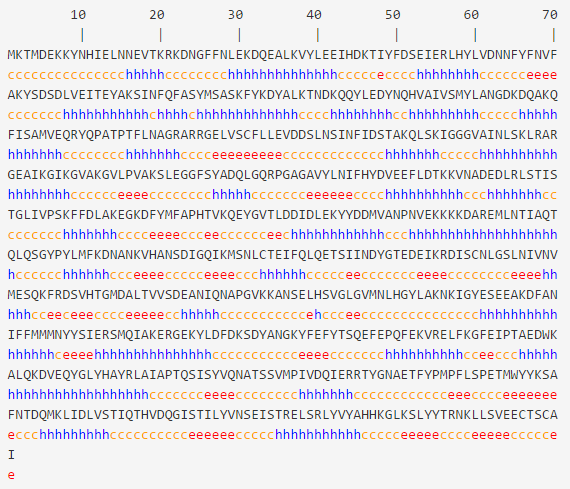


**Figure S3:** Secondary structure of ribonucleotide-diphosphate reductase α was predicted by GOR IV that is composed of alpha helices (291 amino acids), beta sheets (106 amino acids) and random coils (304 amino acids).


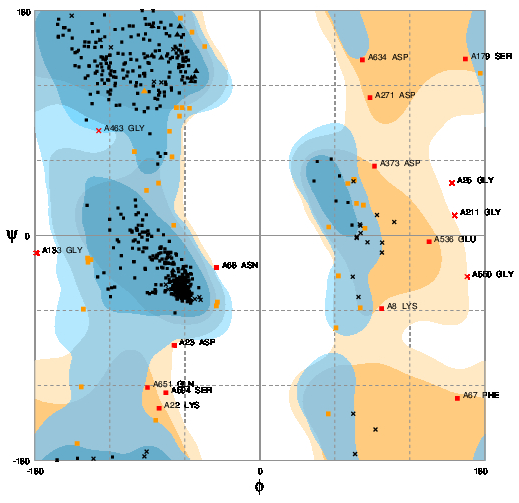


**Figure S4:** Ramachandran plot showed 631 (92.4%) residues in favoured, 32(5.1%) in allowed and 8 (2.5%) in outlier region that demonstrates its good quality structure


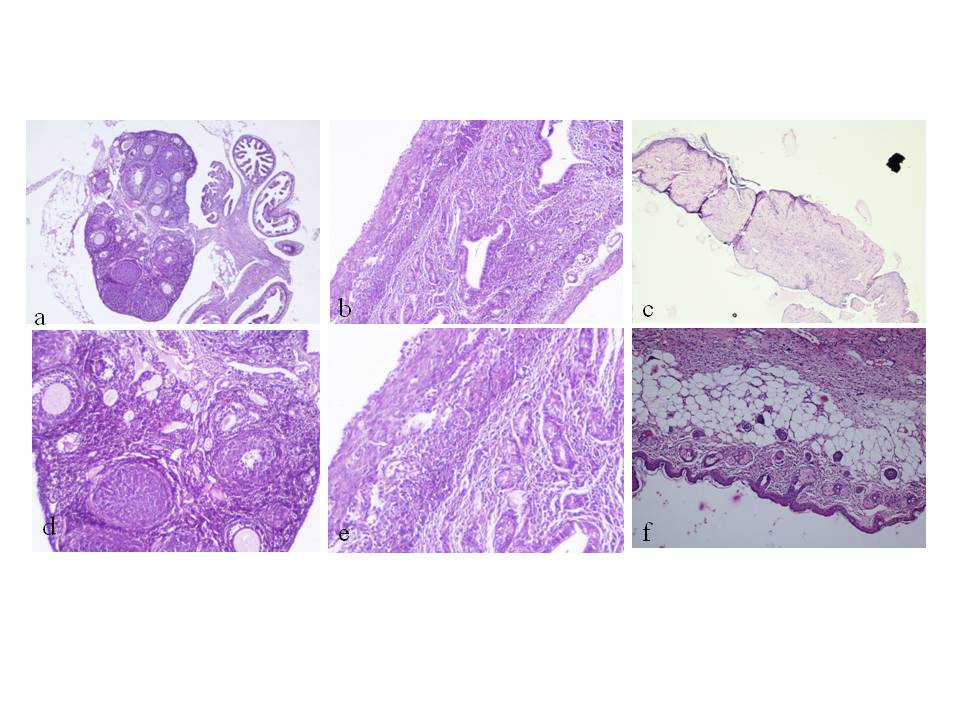


**Figure S5: Histopathological examination of reproductive organs (i.e. ovary, uterus and vagina). Representative photomicrographs showing normal tissue morphology of ovary, uterus, vagina of mice receiving either PBS (a, b, c) or recombinant SAF (d, e, f)**


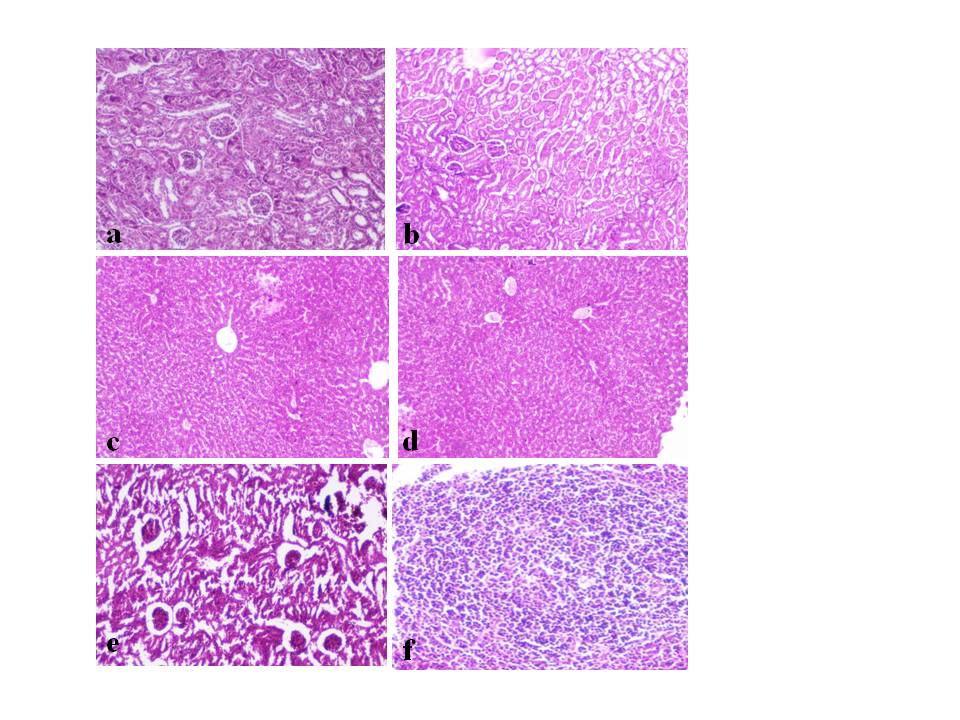


**Figure S6: Histopathological examination of various non reproductive organs of female mice Representative photomicrographs showing normal tissue morphology of Kidney (a,b), liver (c,d), spleen (e,f) of mice receiving either PBS (a, c, e) or recombinant SAF (b, d, f) after 14 days of administration of recombinant SAF**


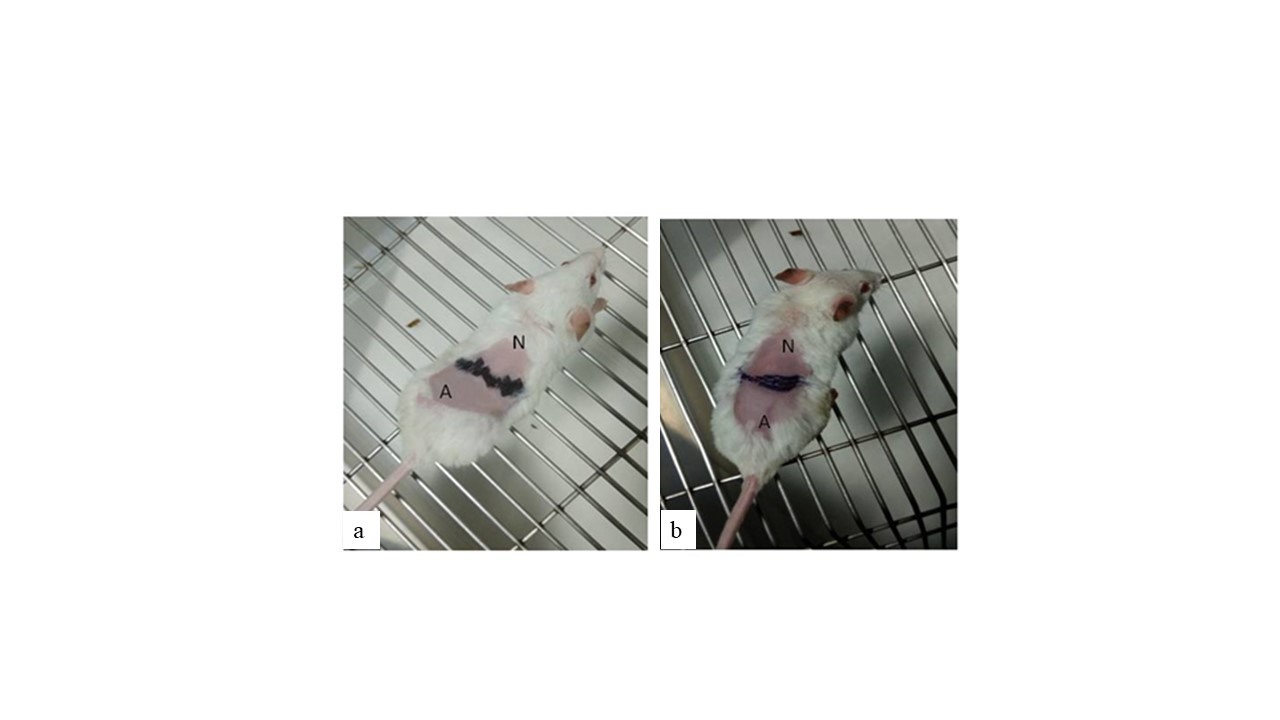


**Figure S7: Evaluation of dermal irritation after application of (a) recombinant SAF (5µg/25μg) in comparison with (b) placebo on abraded (A) and non-abraded (N) test site after 5 day consecutive topical application**


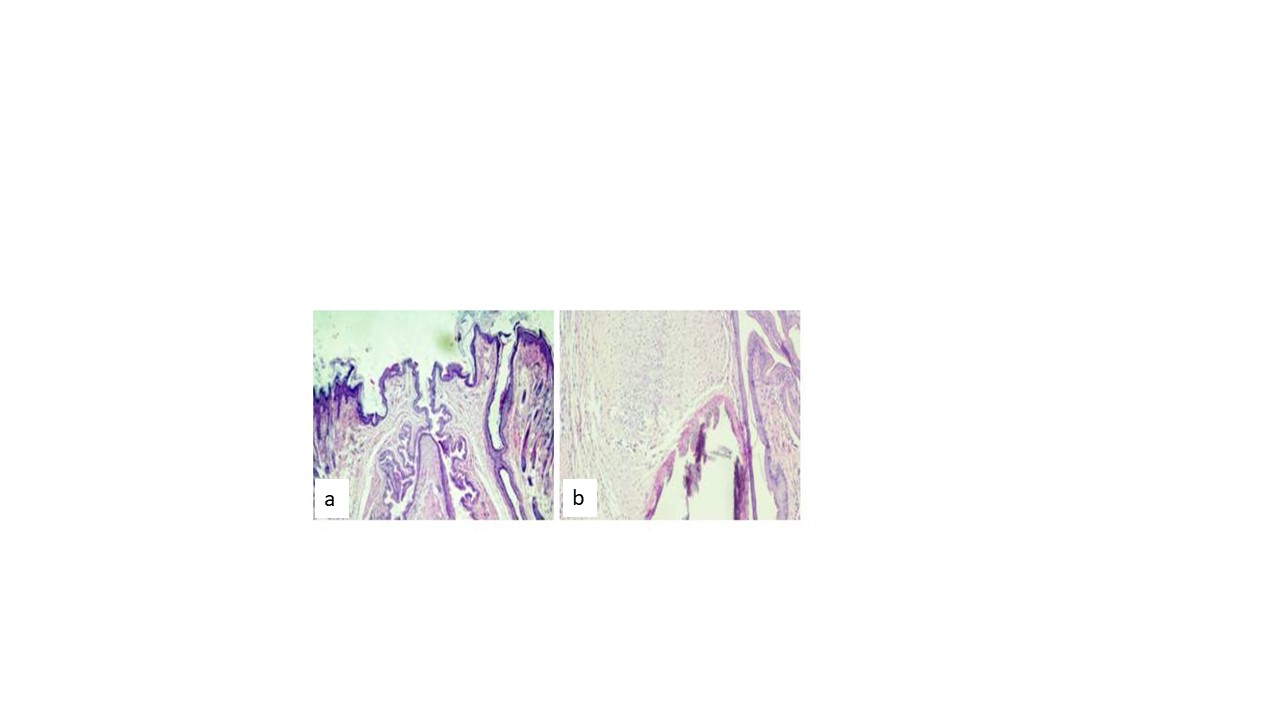


**Figure S8: Penile sections (a) control; (b) treated group (5µg/25μg) showing normal copora spongiosum, intact epithelium without any signs of irritation after 3 consecutive days of application of recombinant SAF**

**Tables**

**Table S1**: Physicochemical properties of Ribonucleotide diphosphate reductase subunit-α

| **S. No.** | **Parameter** | **Ribonucleotide diphosphate reductase subunit-α** |
| --- | --- | --- |
| 1. | NCBI protein ID | AGC91246.1 |
| 2. | Number of aminoacids | 701 |
| 3. | Molecular weight | 80.3kDa |
| 4. | TheroticalpI | 5.19 |
| 5. | Total number of negatively charged residues (Asp + Glu) | 102 |
| 6. | Total number of positively charged residues (Arg + Lys) | 78 |
| 7. | Sub-cellular localization | Cytoplasmic |
| 8. | Signal peptide | No |
| 9. | Grand average of hydropathicity (GRAVY) | -0.447 |
| 10. | Extinction coefficient | 76810M^-1^cm^-1^ |

**Table S2: Tissue somatic indices (TSI %) of various reproductive and nonreproductive tissues after 14-day oral administration of rSAF (1mg/kg body weight) or placebo**

| **Tissue somatic indices (%)** | | |
| --- | --- | --- |
|  | **Test** | **Control** |
| **Liver** | 3.981±0.07 | 4.087±0.06 |
| **Spleen** | 0.667±0.06 | 0.689±0.02 |
| **Kidney** | 1.132±0.03 | 1.115±0.10 |
| **Lungs** | 0.561±0.21 | 0.601±0.08 |
| **Heart** | 0.776±0.11 | 0.746±0.17 |
| **Uterus** | 0.381±0.08 | 0.401±0.05 |
